# Designing for Knowledge Generalization in Medical Device Instructions Among Health Care Professionals: Qualitative Interview and Observation Study

**DOI:** 10.2196/82405

**Published:** 2026-03-06

**Authors:** Qing (Nancy) Xia, Jeremy Opie, Tom Brookshaw, Eloy Erro Barbarin, Ann Blandford, Clare Selden

**Affiliations:** 1 UCL Interaction Centre, University College London, 66-72 Gower Street, London, WC1E 6EA, United Kingdom, 020 3108 7050; 2 Wellcome/EPSRC Centre for Interventional and Surgical Sciences (WEISS), University College London, London, United Kingdom; 3 Institute for Liver and Digestive Health, University College London, London, United Kingdom

**Keywords:** instructional design, interview, think-aloud, observation, health care professionals, human factors engineering, usability studies, medical devices

## Abstract

**Background:**

Instructional documents are crucial for safely operating medical devices. However, few studies have explicitly considered designing instructions for health care professionals (HCPs). Psychological research suggests that advanced learners with prior expertise in a domain are better able to generalize their existing knowledge to new areas compared to those with little prior expertise, resulting in different informational needs.

**Objective:**

We aimed to understand how HCPs learn from and use their existing expertise when interacting with instructions for use to familiarize themselves with a novel medical device. This would allow us to explore how instructional documents could be designed to better accommodate HCPs’ needs as experienced learners with prior knowledge in the domain.

**Methods:**

We conducted our studies centered around a novel liver support system, the HepatiCan and its current instructions for use. We conducted 3 user studies, first using semistructured interviews and think-aloud protocols to understand HCPs’ expectations for the process of setting up the HepatiCan and how training for the use of medical devices typically takes place. We followed this up with an observational study to understand HCPs’ actual experience in practice and corresponding instructional design needs.

**Results:**

Our results showed that HCPs’ prior expertise allowed them to prioritize key areas for attention, but also led them to make assumptions and potentially skim and miss important information. Visual representations and communication were preferred, as well as designs that supported memorization, as this was an essential function of using instruction manuals.

**Conclusions:**

Developers must be aware of what is considered “common” and potentially ignored knowledge within a specific domain in order to clearly emphasize important, safety-relevant information. We also recommend developers focus on using images with attached appendices where possible. This allows advanced users to process information more efficiently without compromising the needs of less familiar users for greater instructional details.

## Introduction

Medical devices play a crucial role in the care and treatment of patients. Ensuring the safety and usability of such devices is therefore essential [1], particularly in mitigating human errors. Human errors account for nearly 70% of all errors leading to preventable adverse medical events [2], such as patient injury or death [3].

One of the primary contributors to medical error is a lack of consideration for human factors and, consequently, poor design that fails to account for user needs [4-6]. Human factors, as defined by the Association for the Advancement of Medical Instrumentation, is “the application of knowledge about human capabilities (physical, sensory, emotional, and intellectual) and limitations, to the design and development of tools, devices, systems, environments, and organizations” [7]. Developing a strong knowledge of users’ needs and capabilities is therefore essential to appropriately cater for and support users in engaging in safe practices, particularly in the context of a medical device.

A key resource for ensuring the usability of a medical device is its accompanying documentation and manuals, that is, instructions for use (IFU). In this paper, the term IFU is broadly used to encompass any document containing information or guidelines that could inform users about how a task or procedure should be executed under normal conditions [8]. This includes standard operating procedures and commercial user manuals. As the functionality of novel medical technologies increases, however, so too does the complexity of user operation and the likelihood of errors during initial setup [9]. IFUs therefore serve as an essential tool for supporting user learning with novel medical devices and should be subject to the same level of rigor and assessment for usability, user comprehension, and learning, as any other component of the system [10]. Indeed, there have been recent demands for greater transparency and clarification for how safety standards and evaluations should be implemented during the development of a novel medical device [11,12]. Our study adds to the growing collection of studies that address these calls to present detailed usability evaluations across a range of medical devices [5,13-16].

IFUs typically communicate information via both text-based and visual representations. Within the field of instructional design, cognitive load theory is commonly applied to help designers explain which types of media and forms of communication may be most beneficial for different types of users in information comprehension and processing [17-19]. Cognitive load theory suggests that individuals’ internal cognitive resources, such as working memory, are finite, and tasks such as learning and processing information place demands, or “cognitive loads,” on these resources [18,20]. Higher cognitive loads can limit an individual’s capacity to attend to important information and are thus more likely to result in lower task performance [21].

The extent to which cognitive load is induced in an individual depends on external factors, such as the design of interaction materials, as well as individual factors, such as an individual’s prior knowledge. This is why it is important to study health care professionals (HCPs) and evaluate their needs in instructional design as distinct from that of layman groups or patients, as HCPs differ significantly in prior domain experience and related medical device knowledge. Previous research suggests that individuals with prior domain experience perform better using procedural, action-oriented, and concise instructions (eg, image only), but worse when using highly detailed, explanatory instructions that are more useful for novices. This phenomenon is known as the “expertise reversal” effect [18,22], and has been observed across many domains and task types ranging from literary interpretation to accounting [18,23,24]. In addition, experienced individuals can encounter negative knowledge transfer [25]. This means they may struggle to disengage from well-established routines and adapt to new knowledge, such as differences in interface designs between similar products [26,27]. Previous research has considered the role of prior knowledge in patients’ use of medical devices [28] and the impact of cognitive workload on HCPs’ preferences for receiving patient feedback [29]. However, there remains a lack of insight into how HCPs themselves navigate unfamiliar medical devices, especially how their extensive training and prior expertise shape their instructional design preferences.

Our study aims to address the issue of evaluating instructional design principles for HCPs [4,30-32]. First, medical device developers often encounter challenges in accessing the perspectives of HCPs during development [33], so it is important for research to provide a clear and practical description of HCPs’ needs. Second, despite the importance of designing devices suited to user needs, there is little advice currently available in regulatory guidelines about specific considerations that are relevant for different user groups (eg, designing for patients or nonmedical staff, compared to trained HCPs) [7,8]. While existing literature has highlighted the importance of supporting HCPs’ training and understanding the challenges these individuals face when navigating complex technologies [34,35], there has been little consideration for the specific characteristics of HCPs which differentiate their needs from other user groups in human factors.

The goal of our research is therefore to investigate how challenges of learning and knowledge transfer may reflect in HCPs’ behaviors in an applied learning setting, and to consider the types of designs which may help to mitigate these issues based on users’ feedback and performance (ie, accuracy or errors made) when following instructions. In the context of medical devices, errors or issues in performance could lead to delays in patient treatment or potentially harmful impacts to patients during treatment [9]. Our research aim is therefore to understand how HCPs learn from, and use their existing expertise when interacting with IFUs to familiarize themselves with a novel medical device.

To address this issue, we use a human-centered approach following the guidelines of the International Electrotechnical Commission [8] to conduct a series of usability studies involving interviews, think-aloud protocols, and observations of HCPs interacting with the IFU for a novel medical device known as the HepatiCan. By conducting these studies, we contribute further insight into the information processing needs of HCPs as experienced learners, how such needs manifest in terms of user behaviors, and provide implications for designing instructions for HCPs.

## Methods

### Case Study: The HepatiCan

HepatiCan is an extracorporeal liver support system that uses cell therapy to treat patients with liver failure. It serves to temporarily replace the functions of the liver, allowing patients’ livers to repair and regenerate. HepatiCan consists of 3 circuits of tubing that are used to circulate blood for treatment. As part of this assembly, sections of circuitry tubing must be connected together using Luer connections and installed into the device’s workstation.

HepatiCan served as an ideal case study for our research purposes because it shares many common features with existing extracorporeal medical devices, such as dialysis machines and apheresis machines, so there was room for an expert in extracorporeal treatment to generalize their knowledge in assembling HepatiCan; and because, as a device still within early developmental stages at the time of the study, there were no existing users whose experience would have otherwise confounded our exploration of “novice” interactions with the IFU. Our paper focuses exclusively on the instructions for assembly for HepatiCan, as this is the first stage of device use before active operation.

Though our focus on the HepatiCan as a case study necessarily means that our sample and corresponding findings are oriented on the experiences of practitioners in extracorporeal treatment, we have striven in this paper to present the needs and patterns that may be applicable to HCPs of varying backgrounds, rather than focusing on specific comments or details which may only be applicable to HepatiCan or the field of extracorporeal treatment.

### Study Design

Part of the original study design aimed to evaluate the usability of the HepatiCan, but in this paper, we present only the information related to the current research aims regarding HCPs’ prior knowledge and instructional needs. The overall study consisted of 2 separate phases, each designed to address a specific research goal. We applied a qualitative descriptive design to capture users’ self-reported experiences in general medical device training as well as their reactions and interactions with the IFU provided in our study, as well as a pragmatic research paradigm to generate actionable insights and recommendations for designers. The first phase consisted of a semistructured interview to understand how HCPs use IFUs in their current workflows and as part of their learning. This helped to contextualize the role of IFUs for HCPs within their wider responsibilities. In the second phase, participants engaged directly with the HepatiCan IFU through a think-aloud and observational study. The think-aloud study had participants read and react to both the IFU document and videos of the IFU procedures carried out by a developer, capturing participants’ perceptions of “work-as-imagined” (WAI) based on simply reading the IFU [36,37]. The observational study had participants interact directly with the HepatiCan and attempt assembly themselves while following the IFU. This allowed us to capture participants’ real-world experiences of following the IFU, or “work-as-done” (WAD) [36,37]. While the arrangement for participants to read through the IFU before interacting with the HepatiCan mirrored how training interventions take place for practitioners, as reflected in the interviews, the goal of this study was not to examine how practitioners respond to training for a novel medical device, but instead the challenges they face in interpreting the IFU specifically. As such, study procedures were designed to place exclusive focus on how participants react to and interact with the IFU.

### Participants

Participants were recruited to this study via invitation from the HepatiCan project leader, as they knew relevant experts with transferable knowledge. Participants had to fulfill 2 criteria to be considered eligible. First, as we were interested in studying “experienced” learners, it was necessary to recruit individuals with existing expertise in areas that were relevant or highly generalizable to the knowledge required in the assembly of HepatiCan’s device components. We therefore looked for participants who had experience in related types of extracorporeal treatment (eg, dialysis), experience or knowledge in hepatology, and/or experience in similar types of extracorporeal systems and medical devices (eg, apheresis machines). This was essential, as HepatiCan is both an extracorporeal device and one that provides hepatological treatments, and thus, in implementation, is intended to be operated by practitioners who work with extracorporeal devices (such as apheresis machines) and those working in hepatological treatments. However, as this is a novel medical device, there are currently no existing user groups. Second, it was essential for us to recruit participants without any prior knowledge or familiarity with the process of assembling the HepatiCan to more accurately replicate the authentic experience of a target user encountering and following the IFU as they would in a real-world context.

In total, 6 participants were invited to take part in the research, though only 3 participants took part in all 3 user studies (Table 1). This limited number was due to the challenges of recruiting participants in the corresponding specialism who had availability during the scheduled timeframe for conducting the study. For the observational study, we required participants to travel to the researcher’s location to engage with HepatiCan, which was a unique prototype and could not be readily transported. Participants were compensated for their time and travel expenses.

**Table 1.** Participants’ backgrounds and participation across all 3 user studies.

Participant ID	Study participation	Background and experience
P1	Think-aloud Observation	PhD graduate in cryobiology
P2	Interview Think-aloud	Training consultant for apheresis machines
P3	Interview Think-aloud Observation	Researcher and clinical consultant specializing in hepatology and nephrology
P4	Interview Think-aloud Observation	Perfusion specialist and clinical operations manager
P5	Interview Think-aloud	Perfusion specialist and clinical research manager
P6	Observation	Clinical apheresis consultant and researcher

These practical considerations limited the outreach and participant sample of our study, which is a common challenge in health care–centered human factors research. However, our use of qualitative methods ensured that we were able to gather rich information and insights from each participant, and our careful recruitment criteria ensured that our findings reflected the experiences of participants who could be considered knowledgeable learners for the context in question.

### Materials

**Figure 1.**
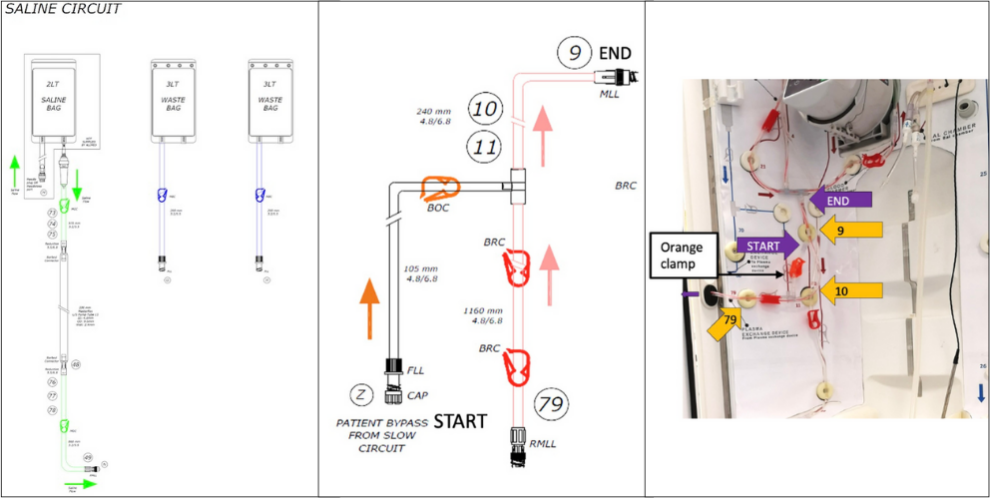
Example of a full circuit diagram schematic (left), a circuit section diagram (right), and an annotated photo for a circuit section.

Our study focused on the IFU for the assembly of the HepatiCan, which was used in both the think-aloud and observational study. This document contained a combination of text and labeled images, with instructions divided across the 3 main circuits of the HepatiCan. Three different types of images were used overall—full circuit diagrams (Figure 1A), circuit section diagrams (Figure 1B), and annotated photos (Figure 1C). Full circuit diagrams depicted labeled engineer schematics of the entirety of HepatiCan’s tubing and inner circuitry. The circuit section diagrams consisted of segments from the full circuit diagram, but zoomed in to show only the relevant circuit section and were presented at the beginning of the corresponding instructions for assembling a particular section. The circuit section diagrams were accompanied by annotated photos of the particular circuit section when assembled to show how the circuit should appear when correctly installed, initially with unmarked colored arrows to denote positions of components or tubing holders. Photos were also used to supplement text instructions to show a consecutive series of steps within a relevant procedure (eg, to demonstrate how 2 locking Luers should be connected).

For the think-aloud, participants were additionally shown video clips of a HepatiCan developer conducting the procedures outlined on the IFU. The videos were silent with the text from the IFUs overlayed as subtitles showing one of the developers assembling the corresponding section. Each clip corresponded to one section of the IFU. There were 4 videos in total, each video corresponding to sections of the IFU relating to assembly of 4 distinct sections of the HepatiCan. The total length of the videos was 12 minutes and 18 seconds. Participants viewed around 10‐30 seconds of the video at a time, representing each step on the IFU.

For the observational study, participants were invited to the laboratory and were asked to assemble the HepatiCan according to the IFU. The materials given to participants included (1) an empty HepatiCan workstation, with the component tubing required for assembly packed in a separate box and labeled as they would be once the device is in clinical use; (2) a printed, color copy of the IFU on single-sided loose pages; and (3) 3 printed and laminated quick reference guides, each on one side of A4, containing the full tubing circuit diagrams for the workstation.

### Data Collection

Both the semistructured interview and think-aloud study were conducted in a single session online using Microsoft Teams. During the interview, the first author used an interview guide to discuss participants’ main priorities when learning from IFUs, their perceptions of how their prior expertise informs their approach to navigating a novel medical device, and how IFUs are generally used during work or training. These discussions were focused on participants’ own experiences and did not involve the HepatiCan or its related documents at this stage. Interviews took around 30 minutes to complete.

Following the interview, participants conducted the think-aloud study. In this study, participants read through the IFU and watched step-by-step video clips of the described assembly process carried out by a HepatiCan developer. Participants verbalized their feedback on the IFU and how well the writing conveyed and aligned with their expectations when watching the video. Feedback on the presentation and layout of the IFU was then implemented following the think-aloud study to improve usability in preparation for the second phase. The think-aloud study took around 1.5‐2 hours to complete.

The observational study was then conducted 2 to 3 weeks after the think-aloud study. Participants were presented with a physical copy of the IFU and were asked to follow the instructions to assemble the HepatiCan device in person. The study was overseen by the first and second authors; 2 developers, 1 in-person and 1 online, were also present throughout the observation. Participants were asked to follow the IFU as they would naturally and were free to ask questions to one of the developers present if they felt it necessary. The in-person developer was told to answer questions and help if asked by the participants, but otherwise not to explicitly intervene except if there was a risk of serious harm or damage. They were also told to identify instances of errors to the researcher (but not to the participant) so that any mistakes which were not noticed or explicitly addressed by the participant could be identified. This was because direct intervention and corrections from developers may otherwise mask the issues and challenges users experience due to instructions from the IFU. The first author observed and recorded participants’ performance in the assembly. Performance was evaluated based on the time taken to complete the assembly procedure, as well as challenges encountered by users, such as errors, hesitation, confusion, and whether they interacted in unexpected ways with the system or required intervention from the developer. The average completion time for the assembly task was around 2 hours across all 4 participants.

Following the observation, the first author conducted a 15-minute follow-up interview exploring (1) overall use experience; (2) preferences and positive interactions with the IFU; (3) challenges and negative interactions with the IFU; (4) use of quick reference materials; (5) addressing key user needs of learnability, ensuring accuracy, and preserving sterility; and (6) suggestions for improvements in the IFU.

### Analysis

In the first phase of the study, the interview transcripts were generated automatically from Microsoft Teams recordings and were manually corrected by the first author. The interview transcripts were then analyzed using inductive thematic analysis to ensure that the themes identified were reflective of participants’ responses [38]. Descriptive codes were initially generated to provide an overview of the transcript data related to the research aims. These codes were then grouped based on relatedness and summarized by higher-level themes.

For the second phase of the study (ie, the think-aloud and observational studies), a spreadsheet was used with columns to represent each section of the IFU evaluated, and each column represented an individual participant’s feedback and queries for each of the respective IFU sections. For the observation, notes were documented and classified using predefined categories to highlight challenges users encountered with using the IFU during assembly. The categories included comments or behaviors related to (1) how participants used and interacted with the IFU; (2) the assembly procedures and how participants conducted each stage of assembly; (3) participants’ experience physically interacting with the hardware, that is, tubing or the workstation itself; (4) errors made by participants while conducting the task (defined as any deviations from the IFU procedures and including errors which were later corrected by the user themselves or the developer); (5) any comments verbalized during the task; and (6) questions and clarifications directed to developers during the assembly process. The follow-up interview was recorded using the first author’s phone and manually transcribed.

During analysis of the data collected in the second phase, descriptive codes informed by the literature were used with a specific focus on topics relevant to the current research aims. This included (1) comments evaluating information appropriateness and relevance to their specific expertise level; (2) comments assessing the advantages and drawbacks of different types of information and presentation formats (eg, visual and text-based); and (3) observed interaction behaviors with the IFU which correspond either to participants’ own descriptions and reported issues with the IFU, or are in line with previous literature documenting challenges experienced users face with specific types of IFUs [17,22].

Our findings are validated through methodological triangulation [39], as different research methods used to explore the same research aim reveal corresponding evidence to support our conclusions. However, there were also instances where participants’ think-aloud reflections and imagined assessment of the IFU differed from their observed behavior and reflections after real-world interactions with the IFU. We report these differences in the “Results” section. In cases of discrepancies, we prioritize recommendations for instructional design based on participants’ observed behaviors rather than their initial think-aloud reactions. This is because individuals’ ability to successfully carry out the necessary procedures had greater implications for risk assessment and patient safety in practice compared to their self-reported perceptions and preferences to specific aspects of the IFU materials.

### Ethical Considerations

The University College London Interaction Centre Research Ethics Committee approved both studies under project number UCLIC/1819/006/BlandfordProgramme-Ethics. The interview and think-aloud activity in Study 1 was conducted and recorded online using Microsoft Teams, which meets the standards of the General Data Protection Regulation and is managed by University College London. Recordings and transcripts were initially stored on OneDrive before being transferred to an encrypted USB drive. All participants were informed of the multistage nature of the study in the information sheet and consent form prior to participation and were given the option to partake in whichever stage they were available for. Participants were paid £35 (US $47.19) for participating in the online study (interview and think-aloud), and £65 (US $87.65) for the in-person session, as well as compensation for travel costs. Transcripts were pseudonymized, and all transcripts and data were stored in an encrypted USB drive.

## Results

### Overview

In the following section, we summarize and report the main findings related to how HCPs use their existing knowledge when interacting with IFUs, and what their corresponding needs may be. We divide the results into 2 sections. The first section reports findings from the semistructured interview study, providing the background and context for understanding how HCPs use IFUs for medical devices in their typical work context. The second section combines results from the think-aloud study and observation. This highlights the key themes identified in how participants initially reacted to the IFU document, both when reading and when applying the document in practice.

### Interview Study

#### IFUs as Initial Learning Tools to Be Discarded With Expertise

The IFU was an essential part of the users’ initial learning process when interacting with a novel medical device. Participants described how IFUs were useful for developing their early conceptual understanding of the device and helping to fill in any gaps in their existing knowledge, such as unfamiliar components within the system. P4 and P5 emphasized that an initial read-through of the IFU was an important part of the process before instructions were implemented and followed in practice.

The operator’s manual is a fundamental tool because you need to go through the operator’s manual before you even start dealing with a new device. [...] Typically when we train on the first time [...] you should have the operator’s manual next to you, which you read before, and then you just follow the manual again in practical terms.

[P5]

Three participants mentioned that the main desired outcome of using the IFU was to achieve a point of familiarization where they no longer needed to use the IFU for assembly or operation. Participants emphasized that this level of expertise was achieved either through intentional practice using the IFU or through a natural result of repetitions over the course of engaging in well-established work routines.

I have the trainee go through it again and again and again, so that it [...] becomes muscle memory.

[P4]

These people are doing the treatments day in, day out. [...] They’re doing it frequently, that’s the key [to memorisation]. If you only do it twice a year, you forget.

[P3]

#### Users Generalize Previous Knowledge to Anticipate Potential Problems

As the IFU provides a preview into the medical device system, use of the IFU allows users to efficiently integrate novel information into their existing knowledge about similar types of devices. When asked, all interviewed participants demonstrated extensive expertise and were able to identify several common patterns across extracorporeal systems, including similar components, terminologies, and procedures. These common features allowed participants to identify areas where previous knowledge from one system could be applied to another unfamiliar system with shared features.

The knowledge base I gained from [named extracorporeal system] I think is transferable to multiple devices. So the principles are generally the same.

[P4]

Notably, participants’ prior experience with similar systems allowed them to anticipate and monitor for commonly occurring problems. This enabled them to formulate generalized assumptions across devices based on shared features and to efficiently prioritize attention toward specific components, assembly procedures, or common errors, even if they have not used a device previously.

If it’s a peristaltic pump or if it’s a centrifugal pump, then you expect different things from it. And I think it’s important because you are pre-emptively looking for potential weaknesses or potential sources of error when you encounter the device for the first time.

[P5]

Beyond the problem of patient safety, consolidated knowledge and familiarity when using a new medical device were also essential because participants often had to fend against external stressors in the environment that were likely to otherwise disrupt their performance or concentration. These stressors included issues such as tension, time pressure, and distractions from colleagues and patients, which may lead users to forget key information that may contribute to errors.

The likelihood of distractions, paired with poor or misleading design choices, can therefore mean that users are liable to make mistakes if they are not consolidated enough in their knowledge.

You have the pressure of having it ready for the therapy. You have different timings that come into play with transplants.

[P4]

Consolidation of knowledge is important, as decisions made based on the design of the device may be vulnerable to assumptions, and users are therefore liable to make mistakes.

So you might think ‘OK, [a tubing line] comes from the patient, go to the first point, then the second point, then the third point, fourth point, and then the dialysis [...] but that’s not how it works. You actually have to put the line through the blood pump first [...] There is a clue [...] but if somebody’s forgotten about that, the line basically looks the same. So it is possible to put it in the wrong way round.

[P3]

#### Focus on Task Completion May Cause Some Users to Ignore IFUs Completely

Though the utility of the IFU and the information it delivers is widely acknowledged, P2 and P4 both noted, based on their experience observing other HCPs in training, that some users may omit the IFU entirely in favor of working directly on the device. This was largely seen as undesirable, with the use of the IFU generally viewed as an essential tool for reducing the likelihood of user errors.

Some people don’t like to follow instructions and [...] they’ll do it [set up] in an illogical order [...] As long as it’s taught properly [...] they generally don’t make mistakes.

[P2]

To counter the tendency to omit the IFU at the beginning of training, P4 described how they would develop information delivered in short form to reduce the perceived time and attention required to use the IFU properly, and encourage further engagement from users.

What I’ve noticed when I’ve given training is people tend not to read operator’s manuals and so we created a quick reference guide, so it’s an easy to follow, ‘do this then do this, then do this.’

[P4]

### Summary of Interview Findings

Overall, participants’ accounts emphasize that the IFU was an essential tool for initial learning. The IFU supported users in having a clear conceptual understanding of the medical device and to avoid errors particularly during the stages of assembly and operation. However, the role of the IFU was also seen to be supplementary, one that should ultimately be discarded due to convenience and the demands of the work environment. As a result of these external influences, HCPs aimed to consolidate their knowledge enough to be able to perform device operations and assembly correctly without reference to the manual. In practice, a potential issue associated with this is that individuals may ignore the IFU from the outset in order to accelerate this learning process and to try and familiarize themselves directly with the device without supplementary support. This may be because participants ultimately build up a general knowledge of similar devices, which allows them to anticipate problems.

### Think-Aloud and Observational Studies

#### Detailed Information Is Initially Useful, but Counterproductive in Practice

Participants’ opinions on the level of detail required for useful instructions shifted from the think-aloud study and the observational study. One recurring example where this debate occurred in the IFU was during a protocol that outlined the assembly process for 2 Luer connections. Luers were connecting points that resembled turn screws, which had to be placed together and turned in a certain direction to ensure that the tubing was securely connected to prevent leakage. Participants described this to be a standard procedure for the assembly of extracorporeal devices, but acknowledged that individuals lacking in expertise may not be familiar with the terms or the process of connecting 2 together. The IFU features an extensive description of how to connect Luers together at each step where Luer connection is required.

During the think-aloud study, participants were generally appreciative of the details and explanatory information incorporated in the Luer protocols, despite their existing familiarity with the steps. P2 and P4 also felt that the details provided were beneficial for minimizing confusion and errors for less experienced individuals than themselves.

This is all very obvious for me. I have experience with transducers [...] So it’s actually really well-written.[...] For me it’s obvious, but for somebody that’s not used to pumps or for somebody who’s not used to this pump...

[P4]

We did note, however, that P1, who had less practical experience with extracorporeal medical devices, had more difficulty interpreting the text during the think-aloud. He was able to clarify the meaning upon watching the video resources.

It’s the first part that I’m struggling to visualise what’s going on... [...] I don’t know. Is it too much writing? [After seeing video] It doesn’t seem as complicated as [the text] is making it out to be. I think the fact that it’s so wordy so closely might make it seem more complicated than it is.

[P1]

During the observational study, however, feedback on the same Luer connection protocols was mainly negative, with the consensus being that such protocols were unnecessary due to the assumed experience of the target users. P6 found that the articulation of the text often did not clearly correspond to how he would have performed the connection protocol according to his own expertise, and that this would cause him to restart connection processes in order to check that his interpretations aligned with his experience. When reflecting on the importance of these instructional texts, he stated:

A perfusionist [...] does not need to be told ‘how to connect a luer lock’ [...] Yes, it does need to be there if you pulled somebody off the street and said ‘follow this’ [...] but you shouldn’t need to tell a healthcare professional.

[P6]

Three participants reflected during the observation that the most important information to convey to a reader was the fact that there was only one possible direction that the Luers could turn for a successful connection. In most cases, participants’ expertise allowed them to deduce this fact despite this information not being clearly emphasized.

Participants suggested that one way of mitigating this issue was to collate potentially extraneous information to be collected in a separate section of the document (eg, a form of glossary) so that users who wanted the extra information could refer back through the document. Participants emphasized, however, that references to different sections of the document should be clearly identified with page numbers and section numbers so that the information could be easily found.

And if you reference other sections of the of the manual [...] I need to know right now where is it? I cannot spend time trying to find it, or going through page by page.

[P5]

#### HCPs May Prioritize Their Own Routines Over Information in IFUs

During our interviews, participants described how HCPs may neglect to follow IFUs during training, despite the importance of the document. We similarly identified this phenomenon during our own observations. During this part of the study, we found that participants may choose not to follow the IFU to prioritize their own specific routines or familiarity with procedures in related devices.

We noted this again in the Luer connection process, which all participants reported to be familiar with prior to the study. Due to the complexity and the perception that the details of the protocol were unnecessary, we found that users, when performing the outlined Luer connection steps in the IFU, often preferred to carry out the procedures based on their own experience, rather than cross-referencing or double-checking with the IFU. Often, users would prefer to deviate from the IFU or even skip large sections of the IFU. P1, P3, and P6 were often seen carrying out the actions instructed in the IFU (eg, “connect the pressure transducer”), completing the action, only then to find more detailed information about the action that they had just performed and how it should be performed. Often, participants would simply assume that the information in the IFU is redundant or repetitive without using further close scrutiny and skim or even skip sections of the IFU to search for the next step. This process of skimming or skipping occasionally resulted in errors and other relevant steps being skipped as well.

In addition to the above behavior, P3 engaged in additional practices that were not mentioned in the IFU—namely, closing all the clamps on the circuitry tubing once they were assembled. Though this did not have a negative impact on the overall assembly process or the outcome of the assembly or operation of the device, it was nevertheless considered a deviation from the IFU as this was not recommended within the text. When asked about this in the interview, P3 clarified that this practice was encouraged in their specific institution when working with similar extracorporeal devices such as dialysis machines. They carried such practices over and applied them to HepatiCan based on their own judgment of appropriateness and relevance, rather than the information conveyed in the IFU.

It’s to do with the dialysis equipment, so I’m very scared of air getting into the system. [...] We always make sure that the clamps are closed initially. The manufacturer may not do that, but we would normally clamp everything [...].

[P3]

#### Images Alone Are Sufficient for Conveying Key Information

Visual representations played a key role in supporting user understanding, both in the think-aloud and in the observation. During the think-aloud, all participants reported using the circuit section diagrams and photos to help them get a sense of the appearance and layout of HepatiCan. However, different images appeared to serve different purposes. Generally, participants described using the circuit section diagrams and their labels for understanding the system flow and identifying specific components and connections. Participants would also refer to the full circuit diagram to understand the relative position of the circuit section they were currently reading about. Photos were frequently used for understanding the relative positions, orientation, state, and appearance of components.

I tend to use the line diagrams for referring to the specific component, and the photos I would say are to back up what I’m thinking. So I look at the diagrams – ‘that makes sense’, and then I look at the photos to see how it’s applied.

[P4]

Images played a similar role in practice as participants performed the assembly task in the observational study. Participants were observed frequently setting pages with the relevant section images aside to reference while handling and orienting tubing. Participants often tried to physically manipulate the tubing, using the marked numbers on tubing sections and components and attempting to match them to position numbers marked on the HepatiCan workstation. This behavior aligned with the procedures participants reported mentally visualizing in the assembly procedure in the semistructured interview, when they were just reading the IFU. Participants would frequently switch between reference images and the real-world equipment, such as the tubing circuits and the workstation.

Well, I pull the tubing out, identify which end was which end based on the schematic [i.e., circuit section diagram], then I looked at the photo, and identified which end was which end on the Workstation, then I looked at the Workstation, and got started because I knew that was the start point.

[P1]

This study also highlighted the importance of the tubing map on the interior of the HepatiCan workstation wall, which was often used in conjunction with the photos and circuit diagrams. Three participants were also observed relying primarily on the images in the IFU during assembly and only referring to the text to double-check the procedural instructions once components were in place. P6 even suggested creating an instruction manual that was predominantly image-based and using less text, stating that this would improve clarity and reduce the ambiguity of text-based instructions.

I found [the wall-map] to actually be quite helpful. But it was obvious, having studied first of all the picture in the [IFU], and then going to it.

[P3]

Using that [IFU], I would like to see more detailed pictures and fewer words. It always makes life easier if there are pictures there showing exactly where something should be connected. Then you can remove a lot of the sentences that start creating ambiguity.

[P6]

Indeed, instances where the images of the IFU did not correspond exactly to the state of the HepatiCan device were immediately detected by some participants and led to confusion and hesitation regarding which section they should be working on as they tried to interpret the discrepancy. These events were predominantly caused by instances in the IFU, which used images from a different section of the circuitry to serve as an example to illustrate a general procedure.

Other discrepancies or ambiguities in the images, which sometimes caused participants to ask for clarification, included a lack of scaling of circuit diagrams to real-life tubing, which meant participants sometimes had difficulty identifying corresponding physical features in the real-life tubing and thus the correct orientation of the tubing.

#### Summary of the Think-Aloud and Observational Studies’ Findings

Participants’ interactions with the IFU revealed that, while detailed information was considered useful and necessary for novices, it can also be counterproductive for experienced practitioners. Our observations revealed that, for our sample, such detail was often unnecessary, difficult to interpret, and disrupted their workflow in practice. It also led some participants to default to tasks according to their prior routines, sometimes ignoring the IFU altogether. Images, however, consistently played an important role in supporting user interpretation. Most participants responded positively to the use of these images and were even allowed to follow the IFU accurately by depending on images alone. Nevertheless, we also acknowledge that there may be limits to the generalizability of these findings beyond assembly tasks and the context of extracorporeal devices, which we reflect on further in the “Discussion” section below.

## Discussion

### Principal Findings

In our study, we identify the key role that IFUs play in allowing experienced learners, such as HCPs, to familiarize themselves with the knowledge necessary to operate a complex medical device. We further establish how HCPs use their existing knowledge to prioritize their attention and time for certain tasks or to prepare for specific risks. However, information that is considered too “obvious” or redundant may negatively impact the performance of experienced users by condoning skipping behaviors that can result in users missing relevant information, such as safety information or confusing the sequence of procedures. Both our observations and participants’ feedback further suggest that visual instructions alone were largely sufficient for HCPs to identify how to proceed with navigating unfamiliar assembly procedures.

In the following sections, we explore the main design implications for HCP-oriented IFUs, namely, the need for memorization, use of prior knowledge, impact of redundant information, and the benefit of visual information, which we summarize in Table 2. Additionally, we present future directions for research and limitations encountered.

**Table 2.** Design implications for HCP^a^-oriented IFUs^b^.

Design implication	Summary
Support memorization	Recognize that users’ end goals are to memorize and reduce the need to reference the IFU where possible Design of medical devices should be intuitive, and allow users to work out procedures with guidance even if IFUs are not available Using clear labels, color coding, mapping, numbering, etc within the device itself to support user learnability
Identify similarities with other devices and distinguish “novel” information	Designers should be familiar with common medical devices used by the target group and recognize that HCP users are not learning to use a novel medical device in isolation, but draw from previous knowledge of related devices for common procedures and to anticipate challenges Instructional design should provide clear cues delineating “common knowledge” and “novel information,” so users can more easily identify relevant information
Reduce redundant information	Avoid redundancies and overrepetition in text and content so users are not encouraged to legitimize behaviors such as skipping, which increases the risk of confusing procedural sequences, delaying learning, and missing relevant information
Recognize the importance of visual information for experienced users	Provide clear visual aids, diagrams, photos, and pictures of procedure sequences to support understanding, clarity, and ease of referencing during usage Supports user goals to minimize reliance on IFUs by reducing the need for slower, text-based information processing for HCPs

a HCP: health care professional.

b IFU: instruction for use.

### Support Memorization

We found that participants relied on instruction documents predominantly for memorizing procedures and for engaging in deliberate practice that was key for developing both their skills and knowledge. One of the benefits of memorization may be linked to speed and convenience, which is often important for HCPs, as they must contend with a variety of competing tasks in their work [40]. Indeed, checking reference materials can slow down performance. In the observational study, participants were often observed actively attempting to minimize interactions with the IFU, with behaviors such as relying only or predominantly on images rather than reading the text; attempting to work out their next steps based on their prior knowledge, intuition, or on the tubing map in the workstation interior; and skipping quickly through steps without referring to other sections in the IFU even when instructed to do so in the text. These findings indicate that, where possible, both the system and instructional designs should prioritize learnability, notably memorization.

Our findings further highlight the need for device designs to be as intuitive as possible, in order to reduce cognitive load and the need for memorization in the first place. Participants’ positive feedback to the system design of HepatiCan demonstrates potentially useful strategies—for example, clear color coding and number coding, along with a wall map of the circuitry layout—which can minimize the need for user learning. Many participants in the observation were able to simply match up components to the correct location during assembly without directly referring to the IFU, which meant they were able to quickly assemble the device. This can increase the likelihood with which individuals carry out correct procedures in cases where they may fail to recall the relevant knowledge.

### Identify Similarities With Other Devices and Distinguish “Novel” Information

Findings from the semistructured interview study suggest that participants were able to apply their existing knowledge across different extracorporeal systems to assist with prioritizing and anticipating potential issues. This is in line with cognitive load theory, outlined above, which suggests that experienced individuals have well-established cognitive processes that can facilitate the processing, memorization, and learning of information more quickly if it relates to the experienced domain, even when the information was originally new or previously unknown to the user [19].

Participants were able to refer to an extensive knowledge base of common patterns across the different medical devices they worked with, including technical knowledge of system components and procedures. This knowledge allowed them to anticipate potential problems and minimize risk by being selective about where and how they directed their attention. In the observational study, participants were also able to use their existing knowledge of Luer connections to deduce the correct procedures without reading the protocol and provided advice on the information that would be most relevant for a user to know. Previous research on the behaviors of expert individuals found that they were also able to fixate more quickly on task-relevant information because they were more quickly able to discern where they should allocate their attention compared to novices [41-43]. The results from our project consolidate these findings and identify how selective attention strategies translate in real-world contexts to support expert users when learning new domain-related information.

It would therefore be beneficial for developers of complex medical systems to be aware of what components are considered “standard” or “common knowledge” within their field, and what components may be completely novel to users and would require extra guidance. Developers could then present all technical components of a medical system and group them according to what is standard and what is novel. This could not only help users understand how the system operates overall [44], but also allow experienced users to quickly identify the information that is relevant to them.

### Reduce Redundant Information

In line with the predictions of the “expertise reversal effect” [24], we observed that more detailed information impeded, rather than supported, performance when users were already familiar with the domain. A key example of this was the repetition of descriptions for recurring protocols, such as Luer connections, which, despite positive feedback during the think-aloud study, were noted to be cumbersome to read and interpret in practice. During feedback in the think-aloud, participants indicated an appreciation for details, but in practice, they were observed engaging in undesirable behaviors such as skimming or even skipping steps in the IFU entirely. In the case of our particular assembly task, as we outline in the “Limitations” section, the consequences of skipping the Luer protocol information were not severe, and errors were usually easily identifiable and recoverable. However, it does highlight the differences between WAI and WAD and provides key insights into some of the conditions that may result in undesirable deviations from WAI.

Importantly, these differences emphasize that it is important for instructional designers not to assume practitioner engagement with IFU. Though in our case, the repetition of the Luer protocol information meant participants were justified in skipping over this information, what was undesirable was the fact that this, in cases, led to users accidentally skipping over genuinely novel or unfamiliar information. Indeed, experts who lapse into automatic or routine behaviors are more vulnerable to errors [26,27,45], and so it is important that designers are thoughtful and careful about repeating information that may appear to legitimize skipping behaviors in practitioners. It should also be acknowledged that participants themselves still indicated an appreciation for extra details and the need to include them to support less experienced individuals. Overall, there were 3 main situations where participants clearly saw the need for additional information (1) to highlight exceptions to the rule, (2) to identify crucial areas for attention, and (3) to inform and clarify genuinely unfamiliar or novel procedures.

Appropriate information delivery in IFUs must therefore strike a balance between providing information that is relevant to users, without giving so much detail that it disrupts comprehension. One way to achieve this balance may be, as some participants suggested in the interview, to provide references that tell users where to find additional information, if deemed necessary. References could either redirect users to previous pages or lead them to a specific glossary or appendix for relevant information. The advantage of this approach is that it can reduce the cognitive load of experienced users, allowing them to focus on information more directly related to their specific task needs, without running the risk of assuming user expertise by excluding information that may be beneficial for less experienced users.

### Recognize the Importance of Visual Information for Experienced Users

Visual information was identified as critical for supporting HCPs to complete tasks. This was made explicit during the in-person studies, with some participants completing the assembly tasks by relying predominantly on images. This supports the findings of Kalyuga et al [17], who ran a study that demonstrated how experts could achieve a similar level of performance using only images, compared to novices using both image and text. This finding suggests that not all information, and certainly not all textual information, is required in IFUs for experienced users such as HCPs to achieve adequate performance and to feel confident about completing a task. This further concurs with the existing literature on IFUs for medical devices, highlighting the effectiveness of visual aids for comprehension [46,47].

However, we acknowledge that in many instances, reliance on visual materials alone can be limited beyond assembly tasks, and a degree of reliance on text-based or alternative media of instructional materials is needed to ensure clarity, especially when communicating the rationale behind different procedures the user may be unfamiliar with. Nevertheless, even in text-based materials, we suggest that visual cues in formatting, such as the use of colors, bolding, or formatting (eg, via bullet points for emphasis), could still be important in supporting experienced users to quickly identify relevant information.

One interesting observation within our study was that participants appeared to prefer images that corresponded more closely to the real-life context of the workstation. Abstract representations, such as the circuit section diagrams, were also useful for understanding the orientation and features of a specific tubing section, although participants ultimately preferred to use photos and the wall map to complete the final assembly. This may be because it was easier for participants to “translate” the positions and features in the photos and wall map to their assembly context, meaning less effort was required for interpretation [48].

### Limitations

We identify 3 key limitations of this study. The first is the relatively limited number of participants recruited, which impacts the representativeness of the sample and the extent to which our findings may be generalized to other practitioners in the field. This was predominantly due to difficulties in accessing the relevant target end users, a prevalent issue for clinical research with medical practitioners [49]. We navigate this by using a variety of in-depth self-report measures and an observational study to collect rich, detailed data to inform our findings.

Another issue as a result of the small sample was that 3 out of the 4 participants studied in the observation took part in the think-aloud study 3 weeks prior as well. Thus, by the time of the observation, there may be some degree of familiarity and anticipation of the procedures with the HepatiCan and the IFU, which may affect how participants interact with the device. However, we argue that as there were at least 2 to 3 weeks between the think-aloud and observation, and no participant had seen the IFU prior to the think-aloud, it was unlikely that any of the participants had become sufficiently familiar with the IFU or the procedures for assembling the HepatiCan to have a significant influence on their assembly performance. Nevertheless, a possible avenue for future work could be to more explicitly explore and manipulate features of the IFU on 2 different groups of naïve users, and to compare performance and feedback within each group in order to establish a causal relationship between designs and desired outcomes.

Finally, we recognize that our recommendations for the contents of IFU design do not consider how external factors such as regulations, legal considerations, and risk management may also impact IFU content, as this can differ widely across medical devices, countries, and institutions. Nevertheless, the findings of our study and recommendations highlight the conditions needed for an “ideal” IFU design where the goal is to optimize learning, efficiency in performance, and minimize the effects of negative knowledge transfer for HCPs. Instructional designers and developers should therefore apply individual discretion and evaluation of their own contexts when considering how to balance these recommendations with other legal and regulatory requirements for IFUs.

### Future Work

Previous works that have explored user needs for instructional designs (eg, [46,50]) typically explore user performance for smaller-scale medical devices, such as asthma inhalers, or nonmedical contexts. Our study examines user responses to instructional designs for a much more time-consuming and complex medical device assembly task. However, we recognize that there may be limitations in the generalizability of our conclusions to other types of device operation tasks. For example, from a procedural perspective, the assembly of the circuits within the medical device involved many repetitions of similar, simple-to-execute behaviors (ie, connecting tubing through Luers and attaching the tubing to the device), which may explain why users skipped through materials or did not feel the need to pay close attention to the IFU, as mistakes made during these procedures were often easy to notice, address, and were relatively inconsequential at this phase of operation.

Future works could therefore focus more on exploring the issue of attention for HCPs’ use of instructions, for example, by testing whether HCPs are more likely to pay close attention to tasks where execution is more complex, and/or where there are greater consequences for patient safety and error recovery, even if the information is already familiar to them. One example of a potentially relevant procedure to study this is device priming, which involves running saline solution through a device in preparation for operation. Priming is a complex task, requiring not only knowledge of the placement and positions of key components (eg, blood warmer and filters), but also domain knowledge regarding the procedures and purpose of priming. This includes knowledge about best practices, monitoring of the priming process, awareness of the status of different components and measures such as pump speed, and the ability to intervene and troubleshoot problems. In such cases, the increase in task complexity and need for monitoring and troubleshooting may mean HCPs are more likely to remain engaged with the IFU. Future work could therefore use the approaches outlined in these studies to explore whether task complexity and higher stakes improve user attention and adherence to the materials.

In addition, we suggest that a focus on providing users with visual cues in instructional design, both for the instructions themselves and for the purpose of locating important information, can be useful. For example, in cases where recommendations in the IFU may differ from participants’ own experiences and practices, experienced users may benefit from clearly visually outlined, emphasized, and easy-to-scan bullet points, colors, and presentations explaining developers’ justifications for their recommendations, so users could quickly locate and compare practices and motivations underlying different practices. However, further research could explore whether such cues are more appropriate or useful for different types of information and task needs. If different types of information could be identified in order of importance and relevance for users, then they could be explicitly identified and extracted during the design of IFUs.

Another potential area for exploration is to consider how the effects of prior expertise may result in challenges or issues over the course of long-term learning and training. Our user studies provide only a cross-sectional examination of user interactions with the IFU in the early stages of learning, but longitudinal methods using quantitative metrics of performance may be able to provide further insights and comparisons between experienced and nonexperienced users in the formation of specific habits, changes in mental workload, and changes in performance over the course of learning [51]. In the case of the HepatiCan, a direct comparison between HCPs and patient users was not feasible, as the device was intended solely for clinical use. However, we believe this line of research can be particularly fruitful in relation to medical devices designed for both clinical and patient users [13], so that future developers may be better able to clarify the differences in instructional needs for both user groups.

### Conclusions

This paper offers important insights into the little-researched field of instructional design for HCPs. Using a combination of interviews and observational methods, we identified that users prioritized memorization and efficiency in task completion. We further identified evidence that the “expertise reversal effect” applies to HCPs in the context of IFU use, and that highly detailed explanations and instructions regarding information already known to the individual can prompt undesirable behaviors such as skimming or skipping. We suggest that developers, in designing IFUs, should focus on identifying common knowledge constructs and terms within a target user base, so that appropriate emphasis can be placed on information more directly relevant to user needs. We further recommend that developers should emphasize conveying information through visual media such as images and diagrams, which not only communicate key positional information but are also succinct and quick for users to process. Through these findings, we aim to provide guidance for developing medical device instructions that are more intuitive, easy to learn, and easy to apply, to support HCPs better in performing their roles.

## Abbreviations

HCP: health care professional
IFU: instructions for use
WAD: work as done
WAI: work as imagined
